# Community health workers in rural India: analysing the opportunities and challenges Accredited Social Health Activists (ASHAs) face in realising their multiple roles

**DOI:** 10.1186/s12960-015-0094-3

**Published:** 2015-12-09

**Authors:** Lipekho Saprii, Esther Richards, Puni Kokho, Sally Theobald

**Affiliations:** Indian Institute of Public Health - Shillong, Public Health Foundation of India, Meghalaya, India; Department of International Public Health, Liverpool School of Tropical Medicine, Liverpool, UK; Regional Resource Centre for North Eastern States, Nagaland, India; Department of International Public Health, REACHOUT Consortium, Liverpool School of Tropical Medicine, Liverpool, UK

**Keywords:** Community health workers, Accredited Social Health Activist, Incentives and disincentives, Roles and responsibilities, India, Qualitative

## Abstract

**Background:**

Globally, there is increasing interest in community health worker’s (CHW) performance; however, there are gaps in the evidence with respect to CHWs’ role in community participation and empowerment. Accredited Social Health Activists (ASHAs), whose roles include social activism, are the key cadre in India’s CHW programme which is designed to improve maternal and child health. In a diverse country like India, there is a need to understand how the ASHA programme operates in different underserved Indian contexts, such as rural Manipur.

**Methods:**

We undertook qualitative research to explore stakeholders’ perceptions and experiences of the ASHA scheme in strengthening maternal health and uncover the opportunities and challenges ASHAs face in realising their multiple roles in rural Manipur, India. Data was collected through in-depth interviews (*n* = 18) and focus group discussions (*n* = 3 FGDs, 18 participants). Participants included ASHAs, key stakeholders and community members. They were purposively sampled based on remoteness of villages and primary health centres to capture diverse and relevant constituencies, as we believed experiences of ASHAs can be shaped by remoteness. Data were analysed using the thematic framework approach.

**Results:**

Findings suggested that ASHAs are mostly understood as link workers. ASHA’s ability to address the immediate needs of rural and marginalised communities meant that they were valued as service providers. The programme is perceived to be beneficial as it improves awareness and behaviour change towards maternal care. However, there are a number of challenges; the selection of ASHAs is influenced by power structures and poor community sensitisation of the ASHA programme presents a major risk to success and sustainability. The primary health centres which ASHAs link to are ill-equipped. Thus, ASHAs experience adverse consequences in their ability to inspire trust and credibility in the community. Small and irregular monetary incentives demotivate ASHAs. Finally, ASHAs had limited knowledge about their role as an ‘activist’ and how to realise this.

**Conclusions:**

ASHAs are valued for their contribution towards maternal health education and for their ability to provide basic biomedical care, but their role as social activists is much less visible as envisioned in the ASHA operational guideline. Access by ASHAs to fair monetary incentives commensurate with effort coupled with the poor functionality of the health system are critical elements limiting the role of ASHAs both within the health system and within communities in rural Manipur.

## Background

Community health workers (CHWs) became prominent with the Alma Ata Declaration in 1978 that recognised primary health care as the key element for improving community health [1]. The World Health Organization characterises CHWs as members of the community, selected by and answerable to the community they work for, and supported by the health system but with shorter training than professional health workers [2–4]. Although these characteristics outline the fundamental relationships informing a CHW’s position, depending on the programme goal, they differ both within and across countries in terms of their roles and responsibilities, recruitment, training and incentives [5].

The literature conceptualises CHW programmes through two different sets of discourses: as service extension workers and as activists for social change. As service extension workers, CHWs are incorporated into the health system to assist doctors and nurses in activities such as immunisation and health promotion [6, 7]. In this sense, they are considered ‘another pair of hands’ [8] as they are helpful in rendering services to underserved populations and they increase the capacity of the health system to address financial and human resource shortages in a resource-poor setting [9]. As activists, CHWs have been conceptualised as social and cultural intermediaries strengthening the interface between the existing health system and the community [10]. In this sense, their role should facilitate community participation and involve engaging in the necessary actions to address the social and cultural barriers that lead to poor health [11].

A Cochrane review [12] on lay health workers who worked amongst low-income communities of wealthy countries or amongst communities from poor countries shows evidence of CHWs’ contribution to reducing child morbidity and mortality, increasing uptake of immunisation and promoting good breast feeding practices. Systematic reviews [13, 14] report that CHWs in low-income countries (such as Bangladesh, Brazil and Nepal) have demonstrated the capacity to improve antenatal, perinatal and post-partum service utilisation and to prevent perinatal and maternal deaths by early recognition and referral of complicated pregnancies. Due to these successes and the increasing recognition of the crisis in human resources for health, deployments of CHWs have become a popular strategy to delivery primary health care at the community level. Many countries in Southeast Asia and Africa including India are planning and implementing CHW programmes at a national scale to strengthen primary health care systems [15].

Several factors have shaped the experiences of community-based health workers, including the type and quality of supervision, level of linkages with health system structures, availability of drugs, clarity of the responsibilities, funding patterns and quality of programme management [16]. Studies have shown that CHWs who come from the communities they serve have higher levels of acceptance from within these communities [17, 18]. Personality traits and skills like communication, motivation, leadership and ability to reach out to community members are also important factors shaping the effectiveness of CHWs [19]. Adequate and appropriate compensation of CHWs has emerged as an important motivating factor for their continued participation in the programme [6, 19–21]. In a performance-based remuneration system, CHWs have to promote the use of health facilities in order to receive incentives. But negative experiences of the community with primary health care may discourage use of health services; it could limit CHWs to earn their incentives [6, 20]. There is a growing body of literature that focuses on technical aspects of CHW programme management such as selection, capacity building, supportive supervision and performance-based incentives [3, 6, 13, 16, 21–24]. However, there are evidence gaps with respect to the extent to which CHWs can be health activists or agents of change, supporting community participation and empowerment which are crucial aspects of health improvement and sustainability [3, 13].

### Accredited Social Health Activist in India

The Accredited Social Health Activist (ASHA) was introduced by the National Rural Health Mission (NRHM) in 2005. They are female cadres of India’s community health worker programme. The primary goal of the ASHA programme is to promote uptake of skilled birth attendance in collaboration with facility-based auxiliary nurse midwives (ANMs) and the Anganwadi worker^1^. Each ASHA is meant to cover a population of 1000 and receive performance- and service-based compensation for facilitating immunisation, referral and escort services for institutional deliveries. Promoting institutional delivery under the national scheme Janani Suraksha Yojana (JSY) is the most common ASHA task which comes with an incentive. JSY is a demand-side financing programme incentivising institutional delivery. ASHAs are paid Rs. 600 (£6.1 approx.) for every woman who is successfully referred for institutional delivery, and the post-partum mother is also entitled to Rs. 700 (£7.1 approx.) [25, 26].

The ASHA programme guidelines envisage three different roles for ASHAs. First, ASHAs are to function as a ‘link worker’ [20], a bridge between the rural and vulnerable population within the health service centres. Second, ASHAs are to function as a ‘service extension worker’, whereby they are trained and provided with a kit that includes commodities such as condoms, oral contraceptive pills, delivery kits and simple life saving drugs including cotrimoxazole and chloroquine [20]. Third, they are conceptualised as ‘health activists in the community who will create awareness on health and its social determinants and mobilize the community towards local health planning and increased utilization and accountability of the existing health services’ ([20], p15).

The national guidelines stipulate that ASHAs are selected from the community they serve and receive 23 days of training in the first year and 12 days of training every subsequent year thereafter. The training curriculum aims to impart the knowledge, skills and attitudes required of an ASHA to effectively perform their roles and responsibilities. Since its implementation in 2005, there have been numerous studies evaluating the ASHA programme [6, 19, 20, 22, 27, 28]. Stakeholders have different interpretations and understandings of the ASHA programme, which has resulted in a state-level variation in programme implementation [20]. As these studies are mainly cross-sectional, they provide limited information on the experiences of ASHAs themselves in realising their different roles as well as the communities they serve. India has huge socio-economic and political diversity, and there is a need to better understand ASHAs’ multiple roles within the many diverse Indian contexts they operate [15]. This study focuses on the state of Manipur which has been relatively under-researched compared to other regions in India.

### Study context

Manipur is a small landlocked state located in the North-East Region of India with an area of 22 327 km^2^[29] and has a population of 2 570 390 [30]. Manipur, though small in size, is unique in respect of its ethnic composition. The Manipur valley comprises of four administrative areas and is mostly inhabited by the dominant Meitei community, whereas the hilly region is administratively divided into five districts and has 33 ethnic communities. In the hills, the two major tribes by current nomenclature are the Nagas and the Kukis. Most of the 33 tribes are characterised as either as Naga or Kuki. Since the 1960s, Manipur has witnessed a series of ethnic and armed conflicts due to separatist movements [31] and demand for homeland and control over resources [32]. This situation has adversely affected socio-economic development, human security and the health situation, including access to and delivery of health services. The population living below the poverty line is 36.89 %, and nearly 52 % of the working population are engaged in agriculture [33]. In rural Manipur, delivery without skilled birth attendance was 52 % in the year 2012–13 [34].

Given these poor indicators of service utilisation, it is important to better understand the role of ASHAs in maternal health promotion. Though national surveys and health management information system data provide quantitative explanation, there is a paucity of literature and evidence that captures the experiences of ASHAs in fulfilling their multiple roles in a context like Manipur which is characterised by geographic, politico-military and cultural uniqueness. Therefore, this qualitative study was undertaken in rural Manipur to explore the perceptions of stakeholders on the ASHA programme and to understand the opportunities and challenges faced by ASHAs in achieving their multiple roles within this particular context and to discuss the implications of this within and beyond India.

## Methods

A descriptive, exploratory qualitative design was deployed in order to gain insight into stakeholders’ perceptions and opinions of the ASHA programme. A qualitative study design was chosen as it is flexible and iterative; qualitative methods are useful in providing explanations and meanings related to the perceptions, experiences and attitudes of the researched within their own context [35, 36].

**Fig. 1:**
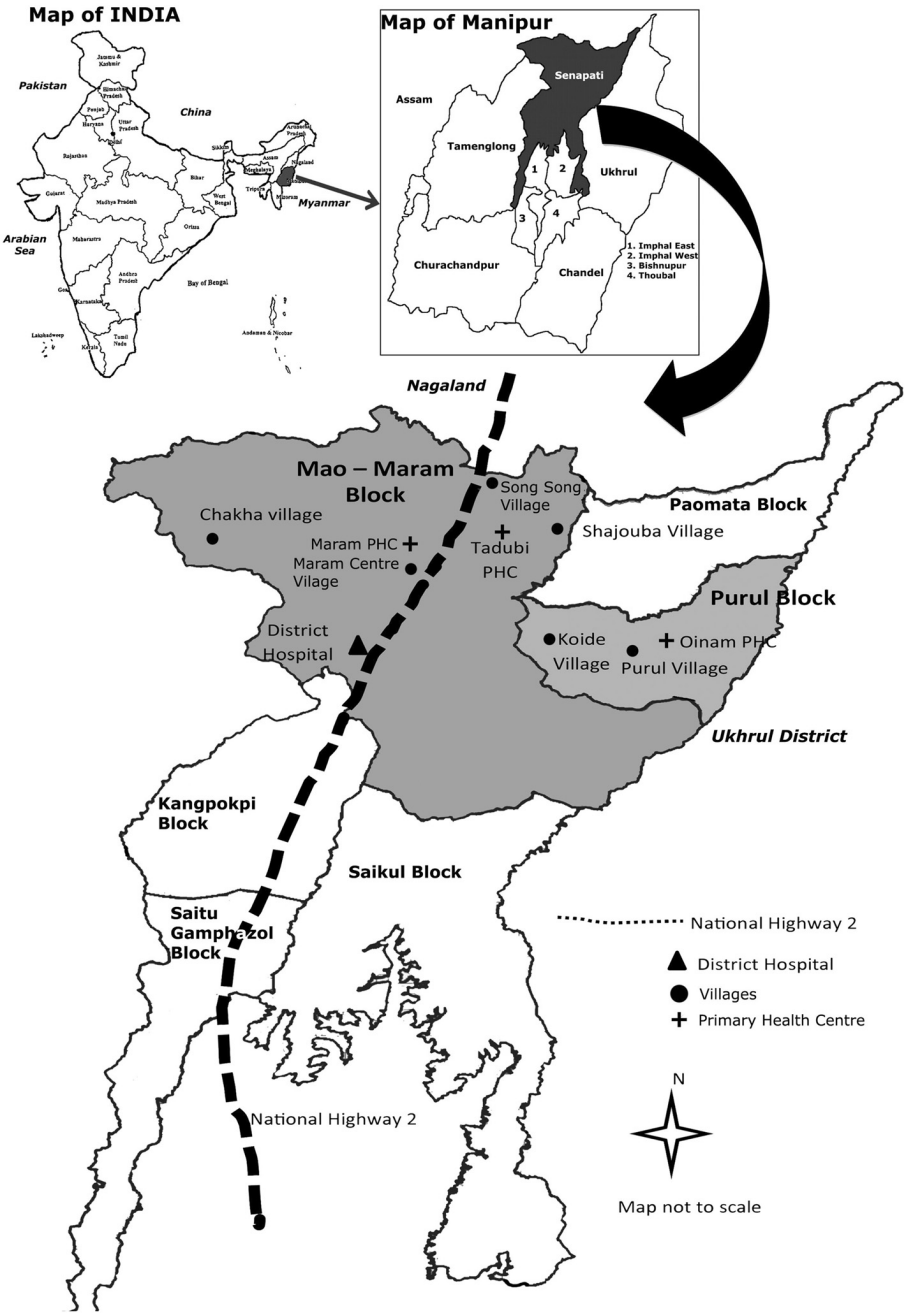
Map of Senapati district and data collection sites

The study was conducted in two administrative blocks (Purul block and Mao-Maram block) of Senapati district in the state of Manipur (Fig. 1). Senapati district was pragmatically selected as the lead researcher (LS) had familiarity with the local dialect and cultural norms and has prior experience of working with the NRHM project in the district. Senapati district is characterised by hilly terrain, and most village settlements are located on the hill top. The villages are widely dispersed with poor roads and communication. It is inhabited by Naga tribes of Mao, Maram and Poumai sharing similar socio-economic and cultural practices. We purposively sampled villages on the basis of their remoteness because we believed that the factors affecting service provision of ASHAs might be affected by remoteness. We sampled seven ASHAs, three post-partum mothers and one Anganwadi worker (AWW) from three remote villages; seven ASHAs, three post-partum mothers and one AWW from three less remote villages; and seven ASHAs from the district headquarter. These villages were selected in consultation with the senior medical officer, and the criteria for remoteness of the village are dependent on the availability of all-weather roads and distance from the highway. We also sampled three doctors and one ANM from three primary health centres (PHCs) that provide service to the sampled villages because they represent a health system that could provide meaningful insights regarding ASHA programmes in the local setting. Three management staff (programme manager, ASHA trainer and ASHA coordinator) were selected from the district programme management unit located at the district headquarter, as they are responsible for the implementation of the ASHA programme.

In-depth interviews and focus group discussions were deployed to explore participants’ perceptions and opinions of the ASHA programme, with the help of a topic guide that was continuously revised based on emerging themes. Eighteen in-depth interviews were conducted (three ASHA, three post-partum mothers who had accessed ASHA services for hospital deliveries and three post-partum mothers who opted for home delivery, three PHC doctors, three management staff, one ANM and two Anganwadi workers) and three focus group discussions (one FGD each of ASHAs representing villages [a] difficult to access PHC [b] easy access to PHC and [c] close to district hospital).

Open-ended questions were used to gather data surrounding the role of ASHAs, the perceived benefits of the ASHA programme and factors that enable or hinder the programme. Written consent was obtained from all the participants, with in-depth interviews (IDIs) lasting for 30–60 min and FGDs lasting between 60 and 80 min in a place and time selected by the participants. The research was led by the first author (LS), a male researcher and social worker from the region with experience of the ASHA programme. As we anticipated that post-partum women may feel discomfort to speak openly on issues pertaining to maternal health to a male researcher, a local female research assistant was recruited and trained to assist in interviews. The interviews were jointly conducted by LS and the research assistant, but she took charge in probing into the pregnancy-related experiences of women. The team ensured that they established good rapport prior to and during the interviews and FGDs.

All interviews were digitally recorded, translated from the local dialect (Mao-Poumai and Manipuri) to English by the research assistant and checked by LS for quality. During and after transcription of data, the study team became familiar with the data by reading the transcripts. Data was managed using NVivo 10. The thematic framework approach was used in data analysis; we first identified recurring and emerging themes which were then clustered into codes and sub-codes [35, 37]. By comparing the codes and sub-codes, a thematic chart was developed in MS Word into hierarchical categories and sub-categories for each theme across all participant groups and used for interpreting the data.

We received ethical approval from the Research Ethics Committee of the Liverpool School of Tropical Medicine, UK, and the Public Health Foundation of India, New Delhi. Written informed consent was obtained from the participants prior to interviews, and participants were given identification numbers to conceal their identities.

### Findings

Findings are presented under two broad categories: (1) role and position of ASHAs in rural Manipur, including as health educator/link worker, service provider and activist and (2) factors influencing ASHAs’ performance. The factors shaping ASHAs’ roles and performance are presented under key themes emerging from the analysis, namely the following: incentives, selection process, training, infrastructure and institutions, gender and tradition and geographical terrain. Illustrative quotes depict key experiences and themes said or implied by multiple respondents.

### Role and position of ASHAs in rural Manipur

#### ASHAs as health educators and link workers

Our analysis of data from rural Manipur reveals that ASHAs are most commonly understood as community health educators and link workers. ASHAs are seen as responsible for disseminating health information and through home visits and counselling motivate women to complete antenatal care and hospital delivery. In our focus group discussion, ASHAs highlighted that:

We [ASHAs] teach the community about nutrition, personal hygiene and sanitation. For example, use of clean cloths and regular change of napkins to prevent infections after delivery (FGD, ASHA [c])

We [ASHAs] are like a bridge between the community and hospital. Since doctors cannot visit all the places, if the need arises, we accompany mothers to services like ANC and blood tests (FGD, ASHA [a])

Deliberating further on the role of ‘educator and link worker’ and their potential benefits, ASHAs and post-partum mothers described a progressive improvement in the level of awareness on the importance of receiving at least three antenatal visits and access to health services. The reasons for improvement is attributed to the presence of an ASHA in the village, as she maintains a list of all eligible couples and pregnant mothers, follows up pregnancy cases, educates and motivates mothers to undergo timely check-ups and escorts women for delivery within hospitals. In our discussion with doctors and nurses, it was stated that ASHAs as link workers, particularly in remote villages, supports the health system in the identification and delivery of services for pregnant mothers and children during outreach programmes such as immunisation and health camps. For example:

Remote villages have poor roads and may take about 5 hours to reach on foot. ASHAs maintain health records of the village; it saves time for the nurse in identification of pregnant women, children and sick people (IDI, PHC Doctor)

#### ASHAs as service providers

In our discussion with ASHAs, it was noted that they were trained and equipped with basic medicine (the ASHA kit) and blood pressure instruments and are able to treat minor illnesses and monitor blood pressure in the community. Most ASHAs viewed themselves as the first port of call for service provision in the community.

The villagers visit us [ASHA] or seek our service irrespective of time to monitor their blood pressure, especially pregnant mothers and elderly people (FGD, ASHA [a])

Most of the ASHAs and post-partum mothers felt that because ASHAs are resident in the village, they are within easy reach of the community and this enables both timely and cost-effective health services for the community. The services provided were cited as health information, minor treatments, monitoring of blood pressure and identification of pregnancy-related complication. Their importance is considered more beneficial particularly in remote areas that are characterised by difficult terrain, poor communication and transportation and high illiteracy.

We can now easily check our BP regularly from her [ASHA] anytime (IDI, Postpartum Mother, Home delivery)

Especially during the seasonal sickness, availability of medicine with ASHAs is very helpful. Our village is about 85 km away from the nearest PHC and with no proper road or transportation, travelling to get medicine would cost us time and money. But with ASHAs, the sickness can be treated on time and saves money (IDI, Postpartum mother, Home delivery)

The importance of ASHAs as service providers was also highlighted by doctors and nurses who viewed the ASHA programme as a strategic opportunity to reduce pressure on the health system as many minor ailments are either treated directly by the ASHA or referred to the ANM stationed in the village or to a public health sub-centre. For example:

There are lots of benefits of having ASHA in the village, especially in the hill districts. Now they are able to make minor treatments. Being able to treat at home by ASHA reduces the pressure on the doctors work load, only those serious cases are referred to hospital. Minor fever and headaches are given paracetamol, even simple antibiotics can be dispensed by them with the proper instruction on the dosage (IDI, PHC Doctor)

#### ASHAs as activists

Analysis of discussion with ASHAs on their role as activists highlighted that they were not aware of what being an activist means or entails. ASHAs highlighted that they were mostly instructed to encourage achieving health targets like immunisation coverage and institutional delivery. In-depth interview with an ASHA trainer revealed that trainings and supervision are mostly focused on institutional delivery and provision of services in the community, with the activist role receiving minimal focus.

We [ASHA trainer] have trained all the ASHAs in modules 1–7; but most of the modules are focused on improving knowledge and skills related to service delivery. Moreover, there is no clear instruction on how ASHAs are to perform as an activist (IDI, ASHA trainer)

As per the NRHM protocol, all ASHAs reported holding the position of secretary in the Village Health Nutrition and Sanitation Committee (VHNSC). However, further probing revealed that ASHAs were not aware of how to manage the committee and their expected role was unclear. In all the FGDs and IDIs, ASHAs explained that they undertook most activities as instructed by the doctor and ANM and that their activities were limited to mobilising mothers for immunisation during the village health and nutrition day and utilisation of VHNSC funds (Rs. 10 000, approx. £115), rather than critical discussion on the status of health in the village and strategies to improve this.

…we [ASHA] only have VHNSC meetings when we get funds from the PHC. We undertake activities like village cleaning, provision of dustbins and provide one or two meals [for nutrition] for old age or single mother (IDI, ASHA)

### Factors influencing ASHAs in performing their roles and responsibilities

#### Influence of monetary incentives

In all the FGDs and IDIs, the monetary incentives provided through JSY was cited as an important factor shaping both the experiences and performance of ASHAs and their relationships with communities and the health system in promoting maternal health. All the ASHAs sampled in the study explained their dependency on the JSY scheme, as it is through this that they receive an acceptable amount of compensation as other tasks are either poorly incentivised or not incentivized at all. As JSY provides the largest amount of incentive for referral of pregnancy cases and escorting women for institutional delivery, ASHAs consider pregnancy cases as their main source of income. For example:

My village is small; there are only few cases of pregnancy. I have limited income compared to villages with huge population (IDI, ASHA)

For every successful delivery at hospital, we receive Rs. 600 [£6.1 approx.]. We work hard to identify all pregnant women in my village, try to motivate them to complete all ANC and delivery in the hospital (IDI, ASHA)

However, doctors and a nurse participating in the IDIs were critical of the incentive-based payment model of the ASHA programme. They perceived that incentive-linked specific activities skew the programme through narrowing ASHAs’ activities to those that are incentivised such as institutional delivery and immunisation and leading to the neglect of other (non-incentivised) activities such as home visits, post-partum care and community mobilisation. For example:

ASHAs’ have a list of work to perform… But since they have limited avenues to earn income, we mostly encourage them to achieve the targets like immunization, hospital delivery, organising monthly village health nutrition day etc.…, so that they could earn some money (IDI, PHC ANM)

On further discussion with ASHAs, all the ASHAs expressed their dissatisfaction with the limited, inconsistent and irregular payment of incentives both to the ASHAs and the mothers who underwent institutional delivery. It was reported that irregular payment negatively affects an ASHA’s ability to execute her role and has negative consequences for her family and the relationship she shares with the community. Recollecting their hardship, ASHAs lamented that the situation is very frustrating. As most ASHAs support their families through agricultural activities, irregular and inconsistent incentive leads to financial instability in the family as they have to manage the basic needs of their families and their children’s education. This has resulted in most ASHAs facing pressure from their families, particularly husbands, to discontinue their role. In such an event, ASHAs reported of engaging themselves in other economically rewarding activities, thus neglecting their professional role. For example:

Since we [ASHA] are mostly cultivators, we often face the dilemma of ‘family responsibility or community work’. Even our husbands disapprove of our work; as it is does not bring any monetary benefit to the family (IDI, ASHA).

Moreover, in all the three FGDs, ASHAs compared their situation to the permanent employment status of other front-line workers, like the Anganwadi worker (AWW), who unlike them receive higher and fixed incentives. Such differences demoralise ASHAs. For example:

Unlike us, the AWW have a fixed salary, whether they work or not they get their salary regularly. What they mostly do is cook food for the children or distribution of ration and some essential medicine … Our workload is much more than the AWW (FGD ASHA [b])

Further discussion with ASHAs highlighted that irregular or non-payment payment creates mistrust with the community they serve, particularly when mothers do not receive the JSY entitlement following hospital delivery. Most ASHAs during FGD and IDIs reported experiences, with community members accusing them of having misappropriated money. Incentives, and their (non- or partial) payment through the JSY system, were seen by both ASHAs and communities as a problematic area, which brought challenges to ASHA-community relationships. For example:

…ANMs are in charge of disbursing the JSY funds entitled to the mothers who had hospital delivery. But when there are delays or non-payment of the incentives, the community, especially mothers accuses us of misappropriating the money. The community members think that ASHAs are government salaried staff. While on the other hand, they [community] do not understand that we are equally dependent on the JSY incentives (FGD, ASHA [b])

#### Selection of ASHAs

Discussion with ASHAs on the selection process showed that they were either nominated by village leaders, requested by the community or that they had applied for the position. Amongst those who had applied for the position, the motivating reasons were source of employment, opportunity to improve their social status and a desire to serve the community.

Since, it is very difficult to find employment, I thought of earning some money by being a volunteer (IDI, ASHA)

We probed about the criteria against which ASHAs were selected or nominated: some of the ASHAs reported that they were nominated by the community or village leaders due to their previous experiences of working as volunteers or had previously occupied leadership roles in the local churches, village youth or women bodies. However, most doctors and nurses interviewed felt that there is ‘biased’ selection of ASHAs. Although ASHAs were nominated by the village community, the final selection of most ASHAs was viewed as based on favouritism and unduly influenced by local leaders:

There are flaws in ASHA selection. Lots of ASHAs are politically appointed in the hope of getting a permanent job like Anganwadi Workers rather than based on criteria of individual capacity to volunteer and sacrifice (IDI, PHC Doctor)

This recruitment process has meant that some ASHAs are not residing in the village or not performing their duties. On enquiring further why underperforming ASHAs are not replaced, doctors and nurse reported that doing so could lead to negative repercussions from the village leaders and others:

…few [ASHAs] never resides in the village nor do they perform their role. But if such practices are reported it will become a problem for me [Doctor] from the village side; including village leaders and local politicians (IDI, PHC Doctor)

#### Training

Nevertheless, when exploring ASHAs’ own experiences of being part of the programme and of the training they received, most ASHAs were happy to continue in the programme as it provides an opportunity to learn and develop their personal knowledge and skills. Indeed, most ASHAs reported that the benefits of their personal growth are directly reflected in their ability to foster healthy behaviour in the family especially in their own child-rearing practices. Moreover, training has helped in improving their knowledge and skills about maternal and child health and other health programmes. They noted a positive correlation between their increased training and the acceptance they experience from the villagers, which eventually enhances their self-esteem and worth:

It makes us feel good, when we share our knowledge on maternal health or child health or any sickness that we know about; they [community] are eager to listen and learn from us. Such community response is very encouraging to us (FGD, ASHA [a])

#### Institutional and infrastructural factors

Discussions with ASHAs revealed that the provision of the ASHA medical kit (which includes drugs and supplies notably paracetamol, chloroquine zinc tablets, iron folic acid tablets, condom, ORS and delivery kit), BP monitor and other non-monetary incentives like torch light, radio and mobile phones were considered essential enablers for ASHAs to be functional and effective in the community. This was more evident amongst those ASHAs residing in remote villages. During a FGD with ASHAs from remote villages, they reported that this equipment makes them a good resource in the village, increases their efficiency and results in a higher acceptance of ASHAs and greater demand for their services.

But now [after BP instrument provided by health department] people, especially pregnant mothers and elderly come to us [ASHAs] for monitoring their blood pressure. They also request us to administer injection; therefore, we are requesting our superiors to train us (FGD, ASHA [a])

In addition, ASHAs living in remote areas recounted that the presence of a local nurse in the village facilitates their role, as services can be accessed at any time. The availability of a trained nurse is particularly beneficial in promoting skilled birth attendance.

Labour pain is unpredictable. It can happen any time. But our sister [nurse] is from the village [local posting in health sub-centre]. She is available anytime if any women is in labour pain or any health emergencies (FGD, ASHA [a])

Reflecting on the challenges experienced by ASHAs in linking mothers to health centres for institutional delivery, both ASHAs and mothers observed that the PHCs were understaffed, ill-equipped and that PHCs do not function 24–7, which brings numerous challenges when PHCs are being promoted (by ASHAs) as a place to give birth. The aversion to government health facilities was more evident and largely due to the poor conditions:

The District hospital has labour rooms but without attached toilets, causing inconvenience to the mother. There is no blood bank and even the minor operation theatre is non –functional. There is no oxygen supply. We can’t even refer cases from peripherals to the district hospital (IDI, PHC Doctor)

#### Gender and tradition

Since ASHAs are situated within a specific socio-cultural and gendered context, they are constantly challenged by existing norms and practices. Discussions with ASHAs highlighted that socially expected gender roles limit their ability to perform their professional role. As a woman, she is expected to fulfil household chores and nurse the children and elderly and failure to do so would attract rebuke from her husband or elders. To fulfil her role as a ‘good’ daughter-in-law, she is also expected to serve her in-laws and to discharge many household duties and other social obligations. One of the ASHAs narrated:

…my husband rebukes me as: What is the point of working for community, when you can’t even take care of your own family? (IDI, ASHA)

According to the majority of ASHAs and doctors, the key challenge in mobilising the community into action is the existing village power structure. Here, gendered norms about women’s roles also proved a barrier. One of the root problems identified was that the women cannot be members of the traditional village and clan council. By tradition, women are excluded and cannot participate in decision-making processes on community matters. As a result, women’s views and needs are often not heard. This has also challenged ASHAs ability to proactively negotiate with the community in setting maternal health as a priority in the village development agenda. One of the doctors narrated:

Since women have no representation in the village council and have little role in decision making with matters related to village; for ASHAs to initiate community action is not very feasible (IDI, PHC Doctor)

Despite women’s limited roles in traditional institutions, doctors and management teams explained that each Naga tribe and village has women’s organisations. While most of these organisations are primarily social-cultural organisation with the backing of the church, others are organised to negotiate peace during armed conflicts or to address alcoholism or drug abuse. But few women’s associations or civil society groups work on, or advocate, for maternal health.

…barely any local health non-governmental organisation exist (IDI, PHC Doctor)

Most of the public organizations, including women associations are more focused on bringing political solution to the contentious Indo-Naga conflict (IDI, Management team)

Finally, the tradition of home delivery was identified as a major obstacle to promoting institutional delivery. Traditionally in the region, delivery is usually conducted at home by an experienced elderly woman. Furthermore, hospital delivery was viewed with suspicion as it was perceived as a ‘last resort’ and most relevant for complicated and extreme cases. Although this trend is changing, these perceptions continue to influence decisions to attend a facility:

People normally go to hospital only when there is complication or obstructed labour. It is traditional to give birth at home (FGD, ASHA [a])

#### Difficult geographical terrain and physical mobility

Discussion with the ASHAs and the research teams’ experience during data collection reveal that location and distance of the village from the health institution shapes the ability of ASHAs to perform their roles. ASHAs whose villages had proper roads and are located close to the national highway did not experience much difficulty in linking the community with the health services. While ASHAs from remote village settlements, usually on the mountain tops, without proper road connectivity and transport system recalled that they need to cover large distances to reach the health facility. Difficult topography and poor transport system restrict the mobility of the ASHA and the community to access health services and also bring challenges to health workers to reach remote communities:

Our village is about 5 hours walking distance from the road [nearest functional PHC is 85 KM]; with no proper transportation pregnant mothers find it difficult to go for ANC check-up (IDI, ASHA)

In addition, all the study participants noted that the ongoing conflict between the state (government agencies) and non-state armed groups and ethnic conflicts between different communities have adversely affected development and health programmes. There are frequent total road closures, curfews and disruption of transport system that restrict physical mobility of service providers and users, including lack of drugs and commodities at the health facility. In one FGD, an ASHA noted:

There were about 75–80 days of road blockade in the first seven months [January to July] of year 2010, organised by different organisation. During such times, no vehicles are allowed to pass. Since, we mostly rely on public transport, it was difficult to motivate or even accompany women for institutional delivery (FGD, ASHA [a])

## Discussion

**Fig. 2:**
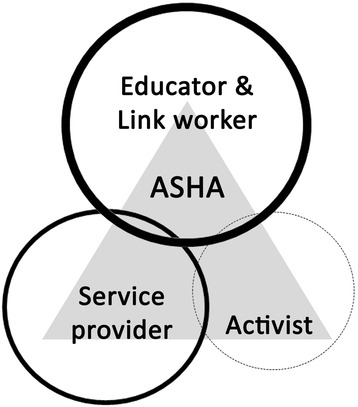
Impression of ASHAs and stakeholders on the role of ASHA

This study has explored the perceptions and experiences of ASHAs in their roles as community health workers and has identified multiple opportunities and challenges ASHAs face in realising their multiple roles. In rural Manipur, the role of the ASHA is predominantly focused on being a link worker or a service provider in the community as illustrated in Fig. 2. Here, the size of the circle represents perceptions about the importance of ASHAs’ different roles while the weight of the line signifies the levels of influence these roles are perceived to have in the community and by the ASHAs.

The ASHA scheme presents a vital opportunity to improve maternal health services through supporting communities to access minor treatments, reducing the pressure on the health system, bringing in-depth health knowledge of the village and facilitating community participation in health programmes [6, 20, 21]. Our findings illustrate how the positioning of ASHAs as an interface between community processes and the public health system is influenced and shaped by several contextual factors in rural Manipur. How ASHAs perceive their multiple roles and their ability to function as a link worker, a service provider and an activist is influenced by these contextual factors, and as a result, some aspects of ASHAs’ roles assume higher importance. For example, performance-based incentives and the nature of supportive supervision encourages ASHAs to focus on promoting biomedical care (in particular institutional delivery) and in achieving targets set by the health facility. While community mobilisation constitutes a core aspect of ASHAs’ roles, lack of adequate support coupled with restrictive cultural and gender norms limit their capacity to engage and negotiate with their communities to create active and empowered citizens capable of claiming ownership of the health programme [20].

Our study confirms the strong association between financial incentives, ASHAs’ performance and the uptake of maternal health services as discussed in other studies [6, 19–21, 26]. The successful provision of financial incentives to ASHAs and mothers to access health services demonstrates the potential to achieve positive changes in health seeking behaviour. However, it is to be noted that ASHAs’ incentivised roles, such as linking pregnant women to ANC and institutional delivery care, receive greater attention which in turn limits ASHAs’ participation in their non-incentivised roles of social activism, community mobilisation, counselling and home visits. The predominance of the role of ASHAs as link workers could be attributed to Janani Suraksha Yojana (JSY), which allows ASHAs to earn a little money for their families. Alternatively, the success of this role may reflect pregnant women being more responsive in general to the incentives provided [19, 23, 38]. While performance-based incentives are clearly desirable and important to motivate ASHAs to participate in the programme, there are multiple implications that require attention from policy makers and programme management. First, anticipation of any financial incentives raises communities’ expectations of getting employment in a government programme, which can compromise the selection process. Second, the focus of ASHAs’ role is limited to performing activities that brings monetary incentives. Third, small incentives coupled with limited avenues to earn money lead to dissatisfaction and constant negative comparisons with other cadres of front-line workers such as Anganwadi. They also encounter challenges in negotiating and realising family expectation and gender norms. Fourth, large CHW programmes (like the ASHA scheme) often lack the resources to pay workers on a regular and reliable basis, which may demoralise and upset ASHAs expecting to receive funds [5, 6, 19, 20]. Several studies on CHWs have indicated that in addition to adequate and timely remuneration for time and energy spent, compensation in kind like housing, child care and recognition on special occasions could facilitate ASHAs to better realise their multiple roles [5, 39]. In short, policy makers should consider consistent, multiple, fair and locally tailored incentives.

While this study was conducted in a unique context of rural Manipur, similar to other studies, we found that nepotism and favouritism are practised in the selection of ASHAs [5]. The influence of nepotism in the selection process may pose multiple challenges to the ASHA programme. First, it endangers the purpose of the CHW programme in achieving inclusive and equitable health, as the needs of the poor and vulnerable population may not be addressed; instead, the relatively privileged community members may benefit most from the ASHA’s attention. Second, an ASHA selected and protected by political leaders may be less motivated and less fearful of dismissal or any punishment for their non-performance. As a result, it may trigger demotivation in other ASHAs, thus leading to poor performance and low delivery of services. Third, literatures highlight the importance of community engagement in the selection of CHWs [7, 19]. However, undue political influence in the ASHA selection may not inspire the confidence of the community, thus rendering the programme fragile and unsustainable from its very inception [3].

Our findings also confirm a range of barriers to community health workers realising their role identified in the broader literature including individual mistrust, the strong tradition of home delivery and poor attitudes of service providers [6, 40, 41]. These barriers impact on the ASHA programme’s capacity to improve health outcomes in rural Manipur. Evidence indicates that users’ perceptions of health services and providers’ behaviour [42], respect for privacy and short waiting times [39], availability of drugs and staff competence [41] are all important determinants for accessing services. In addition, literature also acknowledges that there are challenges in delivery of health care in a conflict setting as lack of security, shortage of skilled health professionals due to migration and harassment from security personnel and non-state armed groups hinder both providers and users in providing and seeking care [43, 44]. Therefore, by tasking ASHAs to promote health services in a conflict zone that are themselves lacking in quality, negatively affects their credibility and trust in the community and restricts their capacity to perform their roles appropriately.

ASHAs as grassroots health advocates are endorsed to mobilise the community, stimulate critical thinking and initiate action to address barriers leading to poor health status [20]. While studies have recognised the importance of CHWs in extending services to marginalised communities and promoting equity [10, 45], our findings add to the literature through revealing multiple challenges to ASHAs realising their activist role. First, ASHAs have no clear understanding of how and what is to be achieved through activism. Second, any actions to address social, economic or institutional barrier are by their nature a political process, which involves a struggle for power and control over resources [46]. Our data raises questions about whether ASHAs are in fact empowered to initiate change, given the patriarchal nature of the societies within which they are embedded and whether there are adequate mechanisms to support their activist role? Third, in a health system that is primarily driven to achieve maternal health targets, is there enough space and will for ASHAs to be activists? When the health system pays incentives for her services, can ASHAs raise their voices against an absentee nurse or doctor? Fourth, ASHAs’ activist role is further limited by inadequate sensitisation of community members on the job description, benefits and commitment of ASHAs and poor engagement of community-based organisations in the programme. Studies have recognised that shared ownership, clarity on the roles and commitments of CHWs, experience of the benefits of CHW programmes and the presence of a supportive community-based organisation are essential elements to sustain community participation in such programmes [5]. However, as mentioned by Ingram et al. [47], there is limited literature available on CHWs as an activist and the processes required to achieve empowered communities. Therefore, the component of CHWs as activists requires further attention.

This study is limited by several factors. First, the sample size is relatively small due to time and logistical constraints, this being a Masters project and had to be conducted within a specific time frame. Furthermore, the study is limited to one district having a homogenous tribal community, a situation which limits generalisability to other non-tribal districts in Manipur. In addition, LS is a member of the community, and having worked with the health department, there are possibilities of interviewer biases. However, LS maintained a field diary and engaged in reflexive processes during data collection and analysis; debriefing with local (PK) and institutional (ST and ER) supervisors were done to mitigate the possible bias. Even though generalisability was not the intention, the present study is unique as it captures the voices and experiences of ASHAs and community in rural Manipur, which has rarely been heard.

## Conclusion

The ASHA programme in India is an ambitious CHW scheme that offers an opportunity for the state government and policy makers and practitioners to improve health. There is need for better understanding of the opportunities and challenges faced by ASHAs in diverse Indian contexts, and this study has highlighted the challenges and realities of this work in rural, conflict-affected Manipur. In the context of rural Manipur, ASHAs were valued for their contribution and promoting opportunities to support maternal health education and ability to provide basic biomedical care, although their role as social activists was considered less substantial. Availability of monetary incentives, fair and commensurate to effort, is an important element for the continued participation of ASHAs. A well-equipped and functional health system can facilitate ASHAs’ ability to perform their roles effectively and at the same time raise their credibility and trust in the community. There is a need to explore how ASHAs may better negotiate their gendered and professional roles in a patriarchal society, such as the socio-cultural context in Manipur and be appropriately supported to drive forward their activist role.

## Footnotes

1 Anganwadi workers are front-line health workers from Integrated Child Development Services under the Ministry of Women and Child Development.
